# Serum calcification propensity is associated with HbA1c in type 2 diabetes mellitus

**DOI:** 10.1136/bmjdrc-2020-002016

**Published:** 2021-02-24

**Authors:** Rik Mencke, Amarens van der Vaart, Andreas Pasch, Geert Harms, Femke Waanders, Henk J G Bilo, Harry van Goor, Jan-Luuk Hillebrands, Peter R van Dijk

**Affiliations:** 1 Department of Pathology and Medical Biology – Division of Pathology, University of Groningen, University Medical Center Groningen, Groningen, The Netherlands; 2 Calciscon AG, Nidau, Switzerland; 3 Department of Internal Medicine, Isala, Zwolle, The Netherlands; 4 Department of Internal Medicine, University of Groningen, University Medical Center Groningen, Groningen, The Netherlands; 5 Diabetes Centre, Isala, Zwolle, The Netherlands

**Keywords:** diabetes mellitus, type 2, calcium, cardiovascular system, hyperglycaemia

## Abstract

**Introduction:**

Serum calcification propensity is emerging as an independent predictor for cardiovascular outcomes in high-risk populations. Calcification propensity can be monitored by the maturation time of calciprotein particles in serum (T_50_ test). A low T_50_ value is an independent determinant of cardiovascular morbidity and mortality in various populations. Aim was to investigate the T_50_ and its relationship to type 2 diabetes mellitus.

**Research design and methods:**

Using nephelometry, serum T_50_ was cross-sectionally measured in 932 stable patients with type 2 diabetes mellitus (55% male) with a median age of 66 (62–75) years, diabetes duration of 6.5 (3.0–10.2) years and hemoglobin A1c (HbA1c) of 49 (44–54) mmol/mol.

**Results:**

Serum T_50_ was normally distributed with a mean value of 261±66 min. In linear regression, serum T_50_ was lower in women and current smokers. A lower T_50_ value was found in patients with a higher HbA1c or higher systolic blood pressure, insulin users and patients with a longer history of diabetes. The association with HbA1c was independent of other determinants in multivariable analysis. There was no association between T_50_ and previous macrovascular events or the presence of microvascular disease.

**Conclusions:**

Serum calcification propensity is independently associated with glycemic control, suggesting that a lower HbA1c may be associated with better cardiovascular outcomes. Retrospective analysis could not establish an association between a history of macrovascular events and T_50_, and prospective studies will have to be performed to address this hypothesis.

**Trial registration number:**

NCT01570140.

**Significance of this study**

**What is already known about this subject?**

- Diabetes mellitus (DM) is accompanied by increased vascular calcifications and an excess cardiovascular morbidity.
- The T_50_ score is a novel functional blood test that quantifies serum calcification propensity.

**What are the new findings?**

- This is the first study to determine associations of T_50_ with parameters of type 2 DM.
- In this large cohort of primary care treated persons with type 2 DM, hemoglobin A1c was significantly associated with T_50_, suggesting that that better glycemic control may correlate with a less pronounced development of vascular calcification.

**How might these results change the focus of research or clinical practice?**

- Although promising, it has to be determined if the T_50_ value has predictive value for cardiovascular disease in the type 2 DM population.

## Objective

Despite intensive glycemic control and adequate management of cardiovascular risk factors, type 2 diabetes mellitus (T2DM) is accompanied by microvascular disease, including retinopathy, nephropathy and neuropathy, and macrovascular disease. Individuals with T2DM are prone to developing vascular calcifications, which considered playing a causal role in the etiology of diabetic complications.^1^

Previously, vascular calcifications were considered a result from passive precipitation of calcium and phosphate. Nowadays, the process of calcification is considered a consequence of a disequilibrium of a between calcification stimulating and inhibiting factors.^2^ Evidence exists that in persons with diabetes this equilibrium is unbalanced, leading to ectopic calcification in the media of the vessel wall, atherosclerotic plaque progression and subsequent cardiovascular events.^1 3–6^ The process of calcification is thought to be (at least partially) mediated by calciprotein particles (CPPs)^7–9^ that naturally circulate in the blood. Primary CPPs contain amorphous calcium phosphate, whereas secondary CPPs contain crystalline calcium phosphate.^10–13^ Secondary CPPs have the capability of inducing calcification of, for example, vascular smooth muscle cells,^7^ so the rate of primary-to-secondary CPP transitioning is viewed to be a measure of the serum anticalcification buffer capacity.

This increased formation and maturation and defective clearance of CPP may be an important novel cardiovascular risk factor (so-called mineral-stress hypothesis).^14^ Indeed, amorphous CPP1 exerted minor cellular responses in macrophage cell lines, while CPP2 appeared to induce oxidative stress and inflammation in macrophages,^15^ and oxidative stress, inflammation, and calcification in primary human aortic smooth muscle cell cultures.^16 17^ The T_50_ serum calcification propensity test has been developed to allow for quantification of the serum anticalcification buffer capacity.^18^ This novel T_50_ test measures in vitro how rapidly CPP2 are formed in a patient blood sample. In other words, the result of the T_50_ test reflects the velocity of calcium phosphate crystallization in blood with lower T_50_ values indicating increased calcification propensity. Results of the T_50_ test have been determined to be an independent mortality predictor in both chronic kidney disease (CKD)^19^ and in renal transplantation patient populations.^20 21^

Because any serum test that can be used reliably to assess vascular calcification would be considered an asset in assessing cardiovascular risk in patients, we aimed to assess the association of the T_50_ test with parameters of T2DM management in a large cohort of stable patients.

## Research design and methods

### Study design and aims

This is a cross-sectional study. Baseline data and blood samples were obtained from the e-VitaDM study and Zwolle Outpatient Diabetes project Integrating Available Care (ZODIAC) study. The e-VitaDM study was designed to assess the feasibility of using an online platform in routine primary healthcare for subjects with T2DM. This study was conducted in general practices that are connected to the Care Group Drenthe in the Drenthe region of the Netherlands (www.hzd.nu). Fifty-two out of the 110 general practices of the Care Group Drenthe agreed to participate; in these practices, approximately 8300 patients with T2DM were treated.

As a prespecified part of the e-VitaDM study, patients were assessed in a long-term follow-up. This prospective arm was nested within the ZODIAC study. Both the e-VitaDM and the ZODIAC study are described in detail elsewhere.^22^ The protocol was also registered on clinicaltrials.gov. All patients gave informed consent.

The primary aim of the present study was to investigate the cross-sectional association between T_50_ and indices of T2DM management, in particular hemoglobin A1c (HbA1c).

### Patients

Patients were recruited during a regular check-up by their (diabetes) practice nurse. Patients were included from May 2012 to September 2014. Patients with T2DM, aged ≥18 years and the general practitioner as main care provider for T2DM were eligible for participation. For the e-VitaDM study, there were no exclusion criteria. A total of 1710 out of 3988 patients, who were asked to participate in the eVita-DM study, gave written informed consent. Of these patients, 730 had no blood samples or were not included in the ZODIAC study and in 48 there was insufficient blood available to measure T_50_. Consequently, the final study sample consisted of 932 patients.

### Measurements

Baseline demographic data included sex, age, duration of diabetes, BMI, alcohol use and smoking habits. Information concerning alcohol use and smoking habits was derived from questionnaires at baseline. Additional medical data were extracted from the diabetes-specific database at the Isala Diabetes Centre. This centre gathers data of primary care treated patients with T2DM in a large part of the Netherlands on a yearly basis to provide benchmark information to general practitioners. This database includes data on physical examination, use of medication, and laboratory blood and urine tests. The following data were extracted: date of diabetes diagnosis, height, weight, diastolic and systolic blood pressure, cholesterol, HbA1c, serum creatinine, urine creatinine, urine albumin, urine creatinine:albumin ratio, the presence of macrovascular complications and microvascular complications.

Macrovascular complications included (a history of) angina pectoris, myocardial infarction, percutaneous transluminal coronary angioplasty, coronary artery bypass grafting, cerebrovascular accident or transient ischemic attack. Microvascular complications included diabetic retinopathy, albuminuria and diabetic peripheral neuropathy. Microalbuminuria was defined as an albumin:creatinine ratio between 2.5–25 mg/mmol in men and 3.5–35 mg/mmol in women. Macroalbuminuria was defined as a ratio higher than 25 mg/mmol and 35 mg/mmol for men and women, respectively.^23^ An ophthalmologist determined presence of diabetic retinopathy biannually. Foot sensibility was tested with 5.07 Semmes-Weinstein monofilaments. Diabetic polyneuropathy was defined as two or more errors in a test of three, at least affecting one foot. Blood glucose lowering therapy was categorized into: dietary measures only, oral blood glucose lowering drugs including metformin, sulfonylurea derivatives, thiazolidinediones and dipeptidyl peptidase-4 inhibitors, and insulin therapy.

At baseline, aliquots of blood samples were stored at −80°C (without thawing) until measurement. Serum T_50_ was measured as described previously.^6^ Briefly, thawing was performed at 4°C for 48 hours, before vortexing and centrifugation. Then, samples were pipetted in triplicate in 384-well plates at 37°C. Supersaturated stock solutions of calcium (35 µL) and phosphate (25 µL), both pH 7.40 at 37°C, were mixed with 40 µL serum, and nephelometry was performed for 600 min in a Nephelostar nephelometer (BMG Labtech, Germany). Non-linear regression analysis was performed on the curves to determine the half-maximal precipitation time.

### Statistical analysis

Normally distributed data are presented as mean±SD, and non-normally distributed data are presented as median (IQR). Means or medians were compared between groups using Student’s t-test or analysis of variance, or the Mann-Whitney U test or Kruskal-Wallis test, as appropriate. Categorical variables were compared using the χ^2^ test or Fisher’s exact test. Normality of variables was assessed using frequency distribution histograms and QQ plots. Univariable linear regression analysis was used to investigate associations between variables that showed a normal distribution of the residuals. Multivariable linear regression analysis was used to investigate associations between a dependent variable and multiple independent covariates, for which a backward regression model was established. Regression models were checked for linearity, homoscedasticity, absence of multicollinearity, independence and normality of errors. A p value <0.05 was considered statistically significant. Univariable logistic regression analysis was used to assess the relationship between a binary outcome dependent variable and an independent covariable. All statistical analyses were performed using SPSS V.24 (IBM, USA).

## Results

Patient baseline characteristics are shown in table 1, both overall and, for descriptive purposes, stratified by T_50_ tertile with the middle tertile ranging from 235 to 293 min. In brief, the population had a median age of 65.8 years (62–75), 54.7% was male, mean diabetes duration was 6.5 (3.0–10.2) years, baseline HbA1c was 49 (44–54) mmol/mol and median (estimated) renal function in this study was 74 (61–86) mL/min/1.73 m^2^. A total of 234 patients (25.1%) had a history of a macrovascular event. A total of 205 patients (30.8%) had a documented microvascular complication. Treatment of diabetes consisted strictly of dietary interventions for 19.5% of patients, whereas 67.5% took oral blood glucose lowering drugs and 13.0% used insulin therapy.

**Table 1:** Baseline characteristics stratified by tertile according to T_50_

	Overall (N=932)	Tertile 1 (<235 min) (N=311)	Tertile 2 (235–293 min) (N=310)	Tertile 3 (>293 min) (N=311)
Age (years)	65.8 (58.5–72.0)	65.7 (57.9–71.7)	65.7 (59.7–72.3)	65.9 (58.7–71.7)
Sex, % male (n)	54.7 (510)	44.7 (139)	53.9 (167)	65.6 (204)
Smoking, % current (n)	17.0 (157)	23.7 (73)	15.2 (47)	12.0 (37)
Alcohol use, % (n)	36.1 (273)	30.1 (78)	38.2 (91)	40.2 (104)
BMI (kg/m²)	29.3 (26.7–33.0)	29.7 (27.1–33.4)	29.1 (26.4–32.8)	29.1 (26.7–32.7)
SBP (mm Hg)	135 (125-144)	136 (124-145)	135 (125-144)	134 (125-141)
DBP (mm Hg)	80 (70-83)	80 (72-84)	76 (70-82)	80 (72-82)
Duration of diabetes (years)	6.5 (3.0–10.2)	6.7 (3.2–10.5)	7.2 (2.9–10.5)	5.9 (2.79.7)
HbA1c (mmol/mol)	49 (44-54)	50 (45-55)	49 (43-54)	48 (44-53)
Total cholesterol (mmol/L)	4.3 (3.7–4.9)	4.2 (3.7–4.9)	4.3 (3.7–4.9)	4.3 (3.7–4.8)
HDL cholesterol (mmol/L)	1.2 (1.0–1.5)	1.3 (1.0–1.5)	1.2 (1.1–1.5)	1.2 (1.0–1.4)
Total cholesterol/HDL ratio	3.4 (2.8–4.2)	3.3 (2.7–4.3)	3.4 (2.8–4.1)	3.5 (2.9–4.3)
LDL cholesterol (mmol/L)	2.3 (1.8–2.8)	2.2 (1.7–2.8)	2.4 (1.8–2.8)	2.3 (1.9–2.9)
Triglycerides (mmol/L)	1.5 (1.1–2.0)	1.5 (1.1–2.1)	1.4 (1.0–2.0)	1.5 (1.0–2.1)
History of macrovascular event, % (n)	25.1 (234)	25.4 (79)	23.5 (73)	26.4 (82)
History of AP, % (n)	7.4 (59)	5.8 (18)	7.1 (22)	9.3 (29)
History of MI, % (n)	8.6 (80)	9.6 (30)	5.8 (18)	10.3 (32)
History of PCI, % (n)	2.6 (24)	3.5 (11)	2.3 (7)	1.9 (6)
History of CABG, % (n)	4.9 (46)	5.5 (17)	4.8 (15)	4.5 (14)
History of TIA, % (n)	3.3 (31)	1.6 (5)	4.5 (14)	3.9 (12)
History of stroke, % (n)	6.1 (57)	5.5 (17)	7.4 (23)	5.5 (17)
History of microvascular event, % (n)	35.2 (293)	35.4 (97)	35.3 (97)	34.9 (99)
History of retinopathy, % (n)	4.6 (38)	7.3 (20)	2.2 (6)	4.3 (12)
History of peripheral neuropathy, % (n)	19.7 (171)	18.5 (53)	20.7 (60)	19.7 (58)
History of albuminuria, % (n)	13.7 (128)	13.8 (43)	13.5 (42)	13.8 (43)
eGFR, mL/min/1.73 m²	74 (61-86)	73 (60-89)	73 (61-85)	75 (63-87)
Albumin/creatinin ratio	0.8 (0.4–1.5)	0.7 (0.4–1.5)	0.8 (0.4–1.5)	1.0 (0.4–1.5)
Diet, % (n)	19.4 (181)	17.2 (53)	19.0 (59)	22.2 (69)
Oral glucose lowering drugs, % (n)	78.1 (728)	79.4 (247)	79.0 (245)	75.9 (236)
Insulin therapy, % (n)	13.0 (121)	16.4 (51)	12.3 (38)	10.3 (32)
Cholesterol-lowering drugs, % (n)	79.6 (741)	77.2 (240)	82.3 (255)	79.4 (246)
Antihypertensive therapy, % (n)	84.7 (788)	84.8 (263)	85.2 (264)	84.2 (261)

Data are presented as percentage (number), mean (SD) or median (IQR).

AP, angina pectoris; BMI, body mass index; CABG, coronary artery bypass grafting; DBP, diastolic blood pressure; eGFR, estimated glomerular filtration rate; HbA1c, hemoglobin A1c; HDL, high-density lipoprotein; LDL, low-density lipoprotein; MI, myocardial infarction; PCI, percutaneous coronary intervention; SBP, systolic blood pressure; TIA, transient ischemic attack.

**Figure 1:**
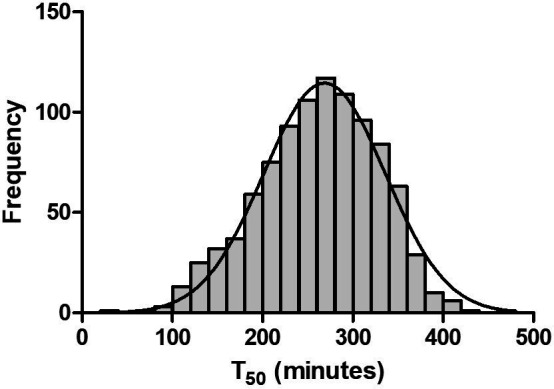
Frequency distribution of serum T_50_. The serum T_50_ values (in minutes) approximate a normal distribution (indicated by the Gaussian curve).

The distribution of serum T_50_ is depicted in figure 1, with a mean value of 261±66 min. While age was not significantly different across tertiles (p=0.664), women had significantly lower T_50_ values (p<0.001). Similarly, a lower T_50_ value was associated with the status of current smoker, while a higher T_50_ value was associated with using alcohol. Furthermore, the tertile with the lowest T_50_ values had the highest Hb1Ac (p<0.001) and more often a history of retinopathy (p=0.016).

**Figure 2:**
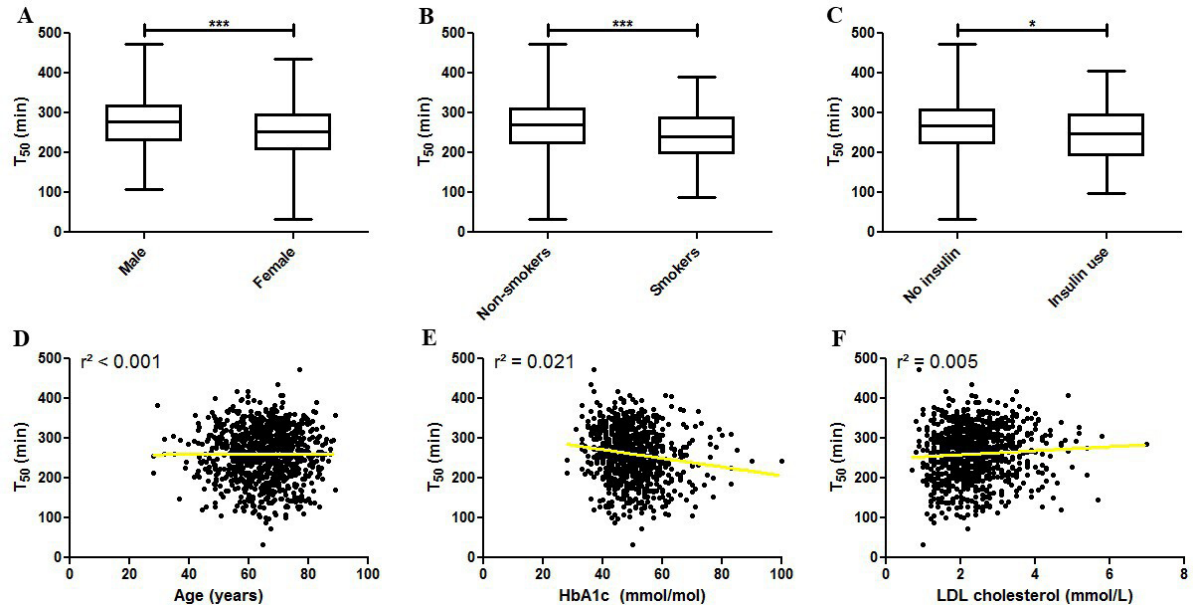
Associations between baseline patient characteristics and serum T_50_. (A) T_50_ is significantly lower in female patients with diabetes. Similarly, T_50_ is decreased in (B) smokers and in (C) patients using insulin. (D) There is no association between age and T_50_. (E) The association between T_50_ and HbA1c is negative and (F) the association between T_50_ and LDL cholesterol is positive. *P<0.05, ***p<0.001. HbA1c, hemoglobin A1c; LDL, low-density lipoprotein.

Determinants for serum calcification propensity were identified using linear regression (table 2). Gender was found to be the strongest determinant of T_50_, with females having a mean T_50_ of 249±65 min, compared with 271±64 min for males (figure 2A, standardized beta −0.171, adjusted R² 0.028, p<0.001). Smoking was found to lower T_50_ (figure 2C), as did a longer disease duration for diabetes. Interestingly, HbA1c was negatively associated with T_50_ as well (figure 2E, standardized (st) beta −0.141, adjusted R² 0.019, p<0.001), whereas low-density lipoprotein (LDL) cholesterol exhibited a significant, positive association (figure 2F). The use of insulin therapy was associated with a lower T_50_ (247±69 min for insulin users vs 263±64 min for non-insulin dependent patients; st. beta −0.083, adjusted R^2^ 0.006, p=0.011; figure 2B). We could not detect an association between the T_50_ and either documented macrovascular or microvascular disease as composite variables. Assessing all conditions separately, only the presence of diabetic retinopathy was found to be significantly associated with T_50_.

**Table 2:** Univariable and multivariable linear regression analysis for determinants of serum T_50_

	Univariable analysis			Multivariable analysis		
	Beta	R²	P value	Beta	Partial R^2^	P value
Age (years)	0.024	0.000	0.911			
Sex	22.435	0.029	<0.001	27.097	0.026	<0.001
Smoking	−22.579	0.017	<0.001	−18.998	0.015	0.003
Alcohol use	8.140	0.003	0.108			
BMI (kg/m²)	−0.492	0.002	0.240			
SBP (mm Hg)	−0.253	0.004	0.068	−0.354	0.004	0.025
DBP (mm Hg)	−0.382	0.003	0.123			
Duration of diabetes (months)	−1.061	0.007	0.011			
HbA1c (mmol/mol)	−1.073	0.020	<0.001	−1.115	0.015	<0.001
Total cholesterol (mmol/L)	1.390	0.000	0.530	−28.616	0.013	<0.001
HDL cholesterol (mmol/L)	−7.930	0.002	0.188			
Total cholesterol/HDL ratio	2.300	0.001	0.248	−9.531	0.006	0.030
LDL cholesterol (mmol/L)	6.323	0.006	0.015	43.982	0.020	<0.001
Triglycerides (mmol/L)	−1.026	0.000	0.644	12.888	0.007	0.009
History of macrovascular event	−3.165	0.000	0.521			
History of AP	9.491	0.001	0.245			
History of MI	−0.971	0.000	0.899			
History of PCI	−19.443	0.002	0.150			
History of CABG	−11.923	0.002	0.227			
History of TIA	14.635	0.002	0.220			
History of stroke	−4.294	0.000	0.631			
Microvascular complication	−2.448	0.000	0.604			
Retinopathy	−23.677	0.006	0.029	−0.075	0.004	0.036
Peripheral neuropathy	−0.557	0.000	0.920			
Albuminuria	0.541	0.000	0.931			
eGFR (mL/min/1.73 m²)	−0.036	0.001	0.416			
Albumin/creatinin ratio	−0.703	0.005	0.037			
Diet, % (N)	6.044	0.001	0.264			
Oral blood glucose lowering drugs, % (N)	−1.057	0.000	0.838			
Insulin therapy, % (N)	−16.035	0.007	0.012			
Cholesterol-lowering drugs, % (N)	4.477	0.001	0.399			
Antihypertensive therapy, % (N)	−4.160	0.001	0.485			

AP, angina pectoris; BMI, body mass index; CABG, coronary artery bypass grafting; DBP, diastolic blood pressure; eGFR, estimated glomerular filtration rate; HbA1c, hemoglobin A1c; HDL, high-density lipoprotein; LDL, low-density lipoprotein; MI, myocardial infarction; PCI, percutaneous coronary intervention; SBP, systolic blood pressure; TIA, transient ischemic attack.

Backward regression was used with all variables to investigate which variables would be significantly associated with T_50_ after correction for covariates. The final model is detailed in table 2. Sex, smoking status, systolic blood pressure, HbA1c, LDL cholesterol, total cholesterol, cholesterol/high-density lipoprotein ratio, and triglycerides all remained significant with the final model explaining 9.4% of the variance in T_50_ (p<0.001).

## Conclusions

In the present, study we explored the association between calcification propensity (measured using the T_50_ test) and indices of T2DM management, in particular HbA1c, in a large population of outpatients. Interestingly, we found that several factors reflecting an unfavorably disease status, namely a higher HbA1c, insulin dependence, and a longer history of diabetes, were all associated with lower T_50_ values. In multivariable analysis, only HbA1c remained significantly associated with T_50_.

This may indicate that long-term glycemic control is a more important determinant for T_50_ than the use of insulin and disease duration. Furthermore, HbA1c remained significantly associated with T_50_ after adjusting for gender, smoking, LDL cholesterol, and systolic blood pressure, identifying HbA1c as an independent determinant. Importantly, poor glycemic control is thought to contribute to the development of vascular calcification,^3^ which is a strong predictor of cardiovascular complications and mortality.^2^ Therefore, the finding that functional serum calcification buffer capacity correlates to markers for glycemic control could be an important step in linking the pathophysiology of diabetes to the pathophysiology of vascular calcification. Given this relationship between better glycemic control and a lower T_50_ value, the T_50_ could prove to be relevant in the follow-up and cardiovascular risk management of patients with T2DM. It is an open question whether, for instance, glycation of factors involved in vascular calcification leads directly to an increased calcification propensity or whether other mechanisms are involved.

Female sex and lower LDL cholesterol levels were associated with lower T_50_ values, indicative of an increased serum calcification propensity. Similarly, the risks of higher LDL cholesterol levels and low calcification propensity may add up to ultimately precipitate a macrovascular event. Serum T_50_ has previously been investigated in renal disease cohorts, including predialysis CKD patients,^19 24^ hemodialysis patients,^25 26^ and renal transplantation patients.^20 21^ Smith *et al*^19^ found that women had a lower T_50_ in CKD stages 3 and 4 like in our study, whereas Pasch *et al*^25^ found that women actually had a higher T_50_ than men (with a difference of about 10 min), in a hemodialysis population. It is possible that the differences between these studies can be explained by the outsize cardiovascular risk, possibly partially CPP mediated, that is conferred by end-stage renal disease, which outweighs the effects of traditional cardiovascular risk factors, which may be more relevant in our non-CKD population and in a prehemodialysis population. On the other hand, Keyzer *et al*^20^ also did not detect an association between T_50_ and gender in a renal transplantation cohort, most of whom had undergone dialysis for over 5 years but who had higher T_50_ values after transplantation than our cohort of patients with diabetes. More studies will be required to elucidate whether associations between gender and calcification propensity, and between serum lipid levels and calcification propensity are disease specific.

Interestingly, serum calcification propensity was not associated with estimated glomerular filtration rate in the present T2DM population but was associated with the albumin/creatinine ratio. This indicates a window between the occurrence of incipient renal damage and the impairment of renal function in which serum T_50_ will start to decrease. Given the pathophysiology of CPPs as a mechanism to buffer calcium and phosphate overload and the relevance of the kidney in regulating phosphate excretion through fibroblast growth factor 23 and Klotho, it is possible that the early disturbances in these factors that occur already during subtle renal injury can rapidly result in an increased serum calcification propensity.

We did not detect an association between serum T_50_ and a history of macrovascular disease or the presence of microvascular disease. The only exception was the presence of diabetic retinopathy, but it had such a low prevalence in our cohort that independent validation in larger or more tailored cohorts will be required to provide reliable answers. It should be noted that macrovascular disease was not assessed prospectively and that a T_50_ measurement at baseline may be a better predictor for future events, rather than reflect past events.

In summary, in this cohort of patients with T2DM, we found that serum calcification propensity was negatively and independently associated with HbA1c, which suggests that better glycemic control may correlate with a less pronounced development of vascular calcification, which ultimately might lead to better cardiovascular outcomes. We were, however, unable to establish an association with the past occurrence of macrovascular disease or with the presence of microvascular disease (with the exception of retinopathy in our univariable linear regression analysis). Prospective studies will be required to elucidate the role of serum calcification propensity and the T_50_ measurement in the vascular burden in diabetes and cardiovascular outcomes.
